# Application of Tuning-ensemble N-Best in Auto-Sklearn for Mammographic Radiomic Analysis for Breast Cancer Prediction

**DOI:** 10.2174/0115734056400080250722024127

**Published:** 2025-07-31

**Authors:** Faikah Awang Ismail, Muhammad Khalis Abdul Karim, Siti Izzatul Akma Zaidon, Kaltham Abdulwahid Noor

**Affiliations:** 1 School of Biology, Faculty of Applied Sciences, Universiti Teknologi MARA, Cawangan Negeri Sembilan, Kampus Kuala Pilah, 72000, Malaysia; 2 Department of Physics, Faculty of Science, Universiti Putra Malaysia, 43400, UPM Serdang, Selangor, Malaysia; 3 Institute for Mathematical Research (INSPEM), Universiti Putra Malaysia, 43400, UPM Serdang, SelangorMalaysia; 4 Dubai Health, Radiology Department, Rashid Hospital, Dubai 00971, United Arab Emirates

**Keywords:** Breast cancer, Machine learning, Auto-Sklearn, Mammography, Radiomic analysis, ACM

## Abstract

**Introduction:**

Breast cancer is a major cause of mortality among women globally. While mammography remains the gold standard for detection, its interpretation is often limited by radiologist variability and the challenge of differentiating benign and malignant lesions. The study explores the use of Auto-Sklearn, an automated machine learning (AutoML) framework, for breast tumor classification based on mammographic radiomic features.

**Methods:**

244 mammographic images were enhanced using Contrast Limited Adaptive Histogram Equalization (CLAHE) and segmented with Active Contour Method (ACM). Thirty-seven radiomic features, including first-order statistics, Gray-Level Co-occurance Matrix (GLCM) texture and shape features were extracted and standardized. Auto-Sklearn was employed to automate model selection, hyperparameter tuning and ensemble construction. The dataset was divided into 80% training and 20% testing set.

**Results:**

The initial Auto-Sklearn model achieved an 88.71% accuracy on the training set and 55.10% on the testing sets. After the resampling strategy was applied, the accuracy for the training set and testing set increased to 95.26% and 76.16%, respectively. The Receiver Operating Curve and Area Under Curve (ROC-AUC) for the standard and resampling strategy of Auto-Sklearn were 0.660 and 0.840, outperforming conventional models, demonstrating its efficiency in automating radiomic classification tasks.

**Discussion:**

The findings underscore Auto-Sklearn’s ability to automate and enhance tumor classification performance using handcrafted radiomic features. Limitations include dataset size and absence of clinical metadata.

**Conclusion:**

This study highlights the application of Auto-Sklearn as a scalable, automated and clinically relevant tool for breast cancer classification using mammographic radiomics.

## 1 INTRODUCTION

Breast cancer is one of the most common causes related to cancer that affects women globally, with 2.3 million new cases reported, and with approximately 12% of all new cancer diagnoses globally [1]. Early detection significantly improves survival rates, and mammography is regarded as the gold standard for breast cancer screening and diagnosis. However, the manual interpretation of mammography continues to pose challenges due to radiologist variability, subjectivity and the difficulty in differentiating between benign and malignant tumors [2, 3].In addition to that, dense breast tissues can obscure tumor detection, leading to false negatives, while some benign cases may be misdiagnosed as malignant, resulting in unnecessary biopsies [4]. These limitations highlight the need for artificial intelligence (AI) and machine learning (ML)-based approaches to improve precision and reduce diagnostic discrepancies [5, 6]. Recent deep learning models, including Capsule Networks and transformer-based segmentation, have demonstrated improvements in diagnostic accuracy and interpretability in disease detection through enhanced Region of Interest (ROI) extraction and feature classification [7, 8]. However, it requires high-end GPU and expertise to be implemented. User-friendly and efficient approaches are needed to make it accessible for wider application and clinical settings.

Radiomic feature extraction helps in quantitative analysis for medical images, which is crucial in diagnosing breast cancer. Denoising and feature enhancement applied before feature extraction help in increasing image quality while preserving structural integrity, especially when multiscale features are required [9-11]. However, some approaches needed expertise in coding, which might be a rarity in clinical settings. Thus, in this study, CLAHE and ACM methods, which are user-friendly, were utilized before feature extraction in this study to achieve optimal quality feature extraction.

Automated Machine Learning (AutoML) has gained attention as a powerful tool for automating model selection, feature extraction, and hyperparameter tuning. Auto-Sklearn, one of the AutoML frameworks, has demonstrated efficiency in optimizing machine learning (ML) models through Bayesian Optimization (BO), meta-learning, and ensemble techniques [12]. Auto-Sklearn enhances classification performance by automating the labor-intensive processes of ML model creation, hence reducing the need for human intervention, which makes it suitable for medical imaging applications. In comparative studies involving other imaging modalities such as brain tumor and Alzheimer’s detection, deep learning models like ResNet, U-Net, attention mechanisms, and more advanced N-Beats have consistently outperformed conventional methods in feature extraction and lesion classification accuracy [7, 13-15]. These findings support the integration of AutoML with robust image preprocessing and segmentation methods to improve outcomes in radiomic breast cancer prediction studies. While previous studies have demonstrated promising advances in medical image classification using deep learning, these approaches primarily focus on manually designed pipelines that require expert knowledge for feature selection, model tuning, and architecture design, which might be challenging to implement in clinical settings. In contrast, this study introduces AutoML- based pipeline that automates not only model selection and hyperparameter tuning, but also ensemble construction, thereby making it more user-friendly for clinical applications.

Due to limitations of manual image processing and reporting, there is an urgent need for an advanced approach to increase the speed of breast cancer detection while optimizing the diagnosis quality *via* AI. In this study, Tuning-Ensemble N-Best in Auto-Sklearn is explored for mammographic radiomic analysis in breast cancer predictions. The methods involve the extraction of radiomic features from mammographic images, including first-order, GLCM texture second order and shape-based features, followed by the application of Auto-Sklearn for tumor classification. Lastly, the performance of standard Auto-Sklearn models with resampling strategies will be evaluated. By leveraging AutoML for mammographic analysis, this study aims to contribute to the development of more efficient, accurate, and automated diagnostic tools for breast cancer detection.

## 2 MATERIALS AND METHODS

### 2.1 Data sources

The sample used in this study was 244 mammogram images with Craniocaudal (CC) and Mediolateral Oblique (MLO) views, extracted from two databases, Curated Breast Imaging Subset of Digital Database for Screening Mammogra-phy and Digital Database for Screening Mammography (CBIS-DDSM) https://www.cancerimagingarchive.net/collection/ cbis-ddsm/.

The samples were categorized as benign or malignant cases based on pathologically confirmed diagnoses made by the experts. After all images were converted into DICOM format, the data were preprocessed and analyzed in MATLAB version R2022a for image enhancement *via* CLAHE and image segmentation *via* ACM. MATLAB programming was used for feature extraction. Later the employment of Auto-Sklearn framework was implemented in Python version 3.9 with Sci-kit Learn for automated model selection, hyperparameter tuning, and model performance evaluation. The study was conducted on a standard desktop computer equipped with an Intel Core i5 (10^th^ generation) processor, 16 GD RAM, and a 512 GB SD.

### 2.2 Image Enhancement and Image Segmentation

Fig. (**1**) illustrates the workflow of the study. The CLAHE was employed to enhance the image contrast, where it utilized the adaptive histogram equalization approach to separate the image into specific parts called “tiles” and performed histogram equalization separately on each tile. The method avoids amplification noise in the image by constraining the amplification contrast in each tile. Each image dataset's contrast of mammogram pictures was enhanced using CLAHE. In this study, the clip limit was set to 0.02 as the most suitable level of contrast, and the number of tiles was set to the default [8 x, 8]. Fig. (**2**) shows the contrast difference between the images prior to and after the application of CLAHE.

After that, the images were enhanced *via* MATLAB R2022a using the *“adapthisteq”* function, the images of the dataset were compared with the images of segmentation that were already segmented by radiologists with manual segmentation. Then, the procedure of segmenting an image starts with the intended ROI on the enhanced mammography images [16, 17]. Later, the ACM technique was applied for the already enhanced images to refine the boundaries of the ROI by iteratively adjusting contour points to fit the tumour shape [18].

In this study, a semi-automatic segmentation approach was used where radiologists initially marked potential tumour regions and ACM refined the segmentation. The ACM energy function consists of three components: elasticity, rigidity, and image gradient attraction, formulated as (**1**):

**Table:** 

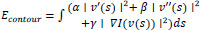	(1)

Where *v*(*s*) represent contour at position (s), *α*controls the elasticity of the contour, *β*maintains the rigidity and *γ*ensures the contour moves towards the image gradient. The number of iterations for ACM was set to 150, ensuring precise tumour boundary detection Fig. (**3**). Illustrates mammography images that are being segmented using ACM.

### 2.3 Feature Extraction

Once the image preprocessing was completed, radiomic features were extracted to quantify tumor characteristics. 37 features were extracted from each mammogram. These features were divided into three categories: Six first-order statistical, 21 GLCM texture second-order, and ten shape features. First-order statistical features encompassed basic intensity descriptors such as mean, variance, skewness, kurtosis, energy, and entropy, which describe the pixel intensity distribution in an image [19].

Second-order texture features using GLCM, which include contrast, correlation, homogeneity, energy, and dissimilarity, provide information about spatial relationships between pixel intensities [20]. Lastly, shape-based features, including area, perimeter, eccentricity, convexity, and solidity, were retrieved to examine the geometric aspects of tumours [21-23]. These features were subsequently normalized and standardized for ML classification. Standardization ensured that all features are on the same scale, preventing bias towards feature values in a range of 0 to 1. Several features are mathematically defined in Eq. (**2**) as follows:

**Table:** 

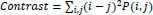	(2)

where *P*(*i*,*j*) is the probability of the pixel intensities i and j occurring together. Furthermore, homogeneity Eq. (**3**):

**Table:** 

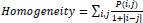	(3)

where values of 1, indicate smoother texture. And finally, the entropy Eq. (**4**)

**Table:** 

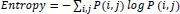	(4)

where, the higher value indicates higher complexity.

### 2.4 Model Training using Auto-Sklearn

The Auto-Sklearn model used in this study was designed to automate the selection, tuning and training of ML models for breast cancer classification. This AutoML utilizes BO, meta-learning and ensemble learning techniques to enhance model performance without requiring manual intervention [17]. It identifies the best-performing classification model based on radiomic features extracted from mammographic images and optimizes hyperparameters for improved accuracy and robustness of the model.

The training process began by uploading the extracted radiomic features obtained from segmented mammogram images. The dataset was later divided into 80% for training and 20% for testing subsets, using stratified sampling to preserve the original class distribution between benign and malignant cases. This approach ensures that both subsets reflect the same proportion of cases as the overall dataset, thereby minimizing potential sampling bias. The Auto-Sklearn library was initialized, and the “*fit*” function was used to train multiple ML models on the dataset. Auto-Sklearn automatically explored a variety of classification algorithms, including Support Vector Machines (SVM), Random Forest (RF), and Extreme Gradient Boosting (XGBoost), to select the best-performing model using cross-validation scores. For SVM, the hyperparameter “*c*” was sampled from a log-uniform distribution with the range 1*e*-3,1*e*3. For Random Forest, the number of estimators (*n_estimators*) was selected from the discrete range of 10 to 1000 and lastly, for XGBoost, learning rates were examined within the range of 0.01 to 1.0. The BO seeks the optimal set hyperparameters, *θ* * by maximizing an objective function (**5**):

**Table:** 

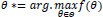	(5)

where *Θ*represent hyperparameter space, and ƒ(θ) is the objective function.

### 2.5 Model Performance Evaluation

The model performance was evaluated *via* the value of accuracy, precision, recall (sensitivity), specificity, F1-score, and the AUC-ROC. Accuracy is defined as the ratio of correct predictions to total predictions. Precision measured the proportion of correctly identified malignant cases among all predicted malignant cases, while recall (sensitivity) quantified the model’s ability to detect actual malignant cases. Below Eq. (**6**) are some mathematical definitions of model evaluation:

**Table:** 

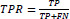	(6)

where TPR is the True Positive Rate, TP, true positive, and FN is false negative. Furthermore, the False Positive Rate (FPR) is defined as Eq. (**7**):

**Table:** 

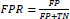	(7)

where FP and TN are known as False Positive and True Negative, respectively. Meanwhile, the AUC-ROC, mathematically defined as Eq (**8**):

**Table:** 

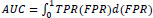	(8)

The specificity score assessed the model’s ability to accurately identify benign cases, thereby maintaining a balanced classification performance. The F1-score, defined as the harmonic mean of precision and recall, was computed to evaluate the balance between false positive and false negative cases.

The ROC curve was plotted to evaluate the true positive rate (sensitivity) about the false positive rate (1-specificity) at varying classification thresholds. The AUC-ROC score offers a comprehensive assessment of the model’s capacity to differentiate between benign and malignant tumours, with values approaching 1.0 signifying enhanced classification efficacy [24].

## 3 RESULTS AND DISCUSSIONS

In this section, the study’s findings were highlighted. The first-order statistical features were found to contribute significantly to model performance, where entropy and variance showed the highest correlation with malignancy, consistent with previous studies that indicated the role of texture irregularity in tumor characterization [25, 26]. Second-order texture features in GLCM, such as contrast and heterogeneity, were found to be strong predictors of malignancy. These features delineate spatial interactions between pixels and offer essential insights into tumor heterogeneity, which is also known as an indicator of cancer aggressiveness [27, 28]. Shape-based features such as eccentricity and convexity further supported classification by capturing geometric differences between benign and malignant tumors, as the latter typically possess uneven shapes with serrated edges, whereas the former generally have smooth, well-defined margins [29, 30].

The performance gap observed between the training accuracy (88.71%) and testing accuracy (55.10%) in the initial Auto-Sklearn model suggests overfitting, where the model performs well on the training data but fails to generalize to unseen data [12]. This anomaly is likely attributed to several factors, notably the limited dataset (244 samples) and high dimensionality of radiomic features (37 features), which may introduce feature redundancy.

To enhance the model performance, the Tuning-Ensemble N-Best approach was implemented, where the top-performing models were combined into an ensemble ML. Additionally, BO was applied to fine-tune hyperparameters, systematically exploring parameter configurations to optimize classification performance. This technique is useful in reducing the number of evaluations required to reach an optimal solution, making Auto-Sklearn computationally efficient. The whole process was repeated 15 times to mitigate the impact of randomness in the model selection and training process, resulting in more consistent and dependable outcomes. This can be seen in Fig. (**4**), where the accuracy score of the model increases after the after-resampling strategy has been applied and repeated 15 times.

Before the resampling, the model tended to favor the majority class (benign cases), leading to poor sensitivity in detecting malignancies. Following resampling, the training accuracy increased to 95.26%, while the testing set accuracy improved to 76.16%, where the recall for malignant cases increased by approximately 18%, improving the model’s ability to correctly identify tumours. This finding aligns with previous studies that emphasize the importance of balancing training datasets to prevent bias in ML models [31, 32].

**Fig. (5):**
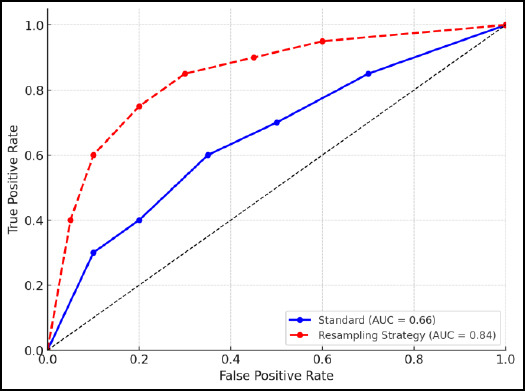
The ROC-AUC graph, blue line indicates performance of standard processing and red line indicates the performance after the application of the resampling strategy for lesion classification.

The enhanced precision and F1-score upon resampling demonstrate that Auto-Sklearn efficiently minimizes false positives and false negatives, making it a promising tool for radiologists in decision-making. After the resampling, the AUC-ROC also improved from 0.660 to 0.840, indicating an enhanced capacity to differentiate between benign and malignant cases for the model (Table **1** and Fig. **5**). From a clinical perspective, this level of discriminative performance is meaningful, as it suggests that the model could potentially assist radiologists in improving early diagnostic accuracy for breast cancer classification. A rise in AUC-ROC signifies a more dependable classification model, with a value approaching 1.0, indicating superior discrimination between two groups [33]. A high AUC-ROC indicates that the model maintains a favorable balance between sensitivity and specificity across different decision thresholds, which is essential in breast cancer screening, where both false negatives (missed malignancies) and false positives (unnecessary biopsies) carry significant consequences. Fig. (**5**) shows the ROC-AUC value for lesion classification increases as compared to the standard processing after application of the resampling strategy, where the x-axis represents False Negative Rate and the y-axis represents True Positive Rate.

A comparative analysis indicates that Auto-Sklearn surpassed conventional models in terms of adaptability and automated hyperparameter tuning. The SVM model achieved an accuracy of 72.3%, RF scored 74.1% and XGBoost obtained 75.6%, whereas Auto-Sklearn with resampling reached 76.16%, underscoring the benefits of automated model selection and optimization. This is consistent with findings from previous studies, where AutoML frameworks demonstrated improved accuracy and efficiency relative to manually optimized ML models [12]. The ability of Auto-Sklearn to automatically select the best-performing algorithms and optimize hyperparameters significantly reduces the time and expertise required for model development, making it effective for medical imaging applications in clinical settings.

The computational efficiency of Auto-Sklearn was also evaluated. The total computation time for Auto-Sklearn training and optimization was significantly lower compared to manual hyperparameter tuning in conventional models. In our study, Auto-Sklearn was able to complete the optimization process within 30-40 minutes, as it enabled fewer steps for ML processing for our dataset; however, it might not be applicable for studies involving larger datasets and more complicated processing. Previous studies demonstrate the necessity for more powerful GPUs and extended training times for dataset processing, particularly when dealing with huge datasets that encompass complex architecture and multi-modal data [14, 34].

While Auto-Sklearn includes built-in resampling and ensemble strategies to mitigate overfitting, it might be insufficient in small-scale radiomic studies. To further lower the risk of overfitting, future work could incorporate explicit feature selection techniques such as Recursive Feature Elimination (RFE) and Principal Component Analysis (PCA), and regularization techniques.

Despite the promising results, the model exhibited several misclassifications that warrant further analysis. Specifically, false-positive cases, which are often associated with benign tumors in dense breast tissue, may appear irregular or heterogeneous in texture, leading the model to misclassify them as malignant. Conversely, some malignant tumors with relatively smooth boundaries were predicted as benign, likely due to their lower texture complexity or homogeneous shape. However, by relying solely on handcrafted radiomic features, some subtle morphological or contextual cues present in mammographic images might be undetected.

Additionally, a small dataset (n=244) and absence of complementary information such as clinical metadata may reduce the model’s ability to differentiate borderline cases. The relevance of this study’s findings can be strengthened by external validation, as it might help to train the models in the clinical setting. These challenges indicate the need to integrate deep learning approaches such as Convolutional Neural Networks (CNNs), with AutoML, which could further improve the classification performance. CNNs excellence in feature extraction may boost AutoML by enhancing spatial feature representation. The hybrid methods, such as ResNet and DenseNet could play a role as feature extractors *via* transfer learning, where deep features derived from mammographic images become an input into the AutoML pipeline for model selection and optimization. Previous studies suggested transformer-based model approaches, especially when the diagnosis involved multi-layer image processing and multi-imaging modalities, as they provide higher model performance compared to conventional deep learning approaches [7, 14, 34]. This approach can be considered in the future for further studies, especially in breast cancer diagnosis.

## 4 STUDY LIMITATIONS

While the findings of this study are promising and demonstrate the potential of Auto-Sklearn for radiomic-based breast cancer classification, several limitations must be acknowledged. The sample size (n=244), though acceptable for an exploratory study, may restrict the generalizability of the model to larger, more diverse clinical populations. The utilization of publicly accessible CBIS-DDSM data, despite its standardization, may not entirely capture the diversity in actual clinical settings, thereby leading to selection bias. Moreover, the reliance on handcrafted radiomic features may not capture the full complexity of tumor heterogeneity, especially in cases involving dense breast tissue or subtle malignancies. As this is an exploratory study, the absence of external validation and the exclusion of clinical metadata may have reduced the model’s ability to handle borderline or ambiguous cases. Nevertheless, the study provides a solid foundation for the use of AutoML in breast cancer diagnosis.

## CONCLUSION

This study demonstrates the feasibility and effectiveness of integrating Auto-Sklearn, with radiomic feature analysis for the automated classification of breast tumors using mammographic images. By incorporating user-friendly preprocessing methods such as CLAHE and ACM for image enhancement and segmentation, the study successfully extracted 37 radiomic features and achieved a post-resampling testing accuracy of 76.16% with an AUC-ROC of 0. 840. These findings highlight AutoML’s capacity to automate model development and improve reproducibility in medical imaging workflows, particularly in clinical settings with limited technical expertise. The contribution of this study lies in its development of a user-friendly, scalable, and effective diagnostic pipeline that minimizes manual intervention while preserving clinical relevance. Future studies should focus on incorporating larger, multi-institutional datasets and external validation to enhance model generalizability. Moreover, combining AutoML with deep learning approaches such as CNNs and transformer-based architectures may enable the integration of handcrafted and deep features, further improving classification performance in real clinical settings.

## Figures and Tables

**Fig. (1):**
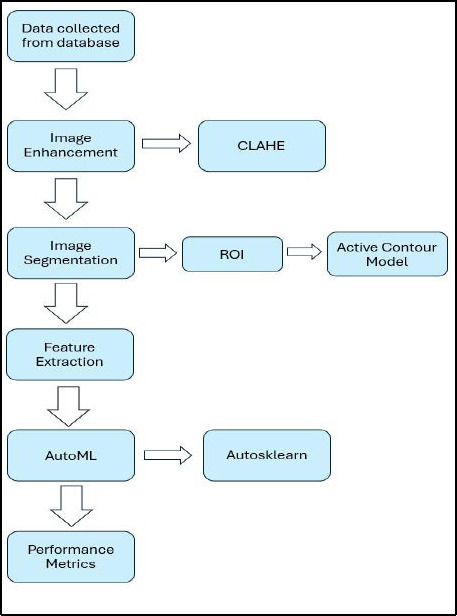
Workflow for study.

**Fig. (2):**
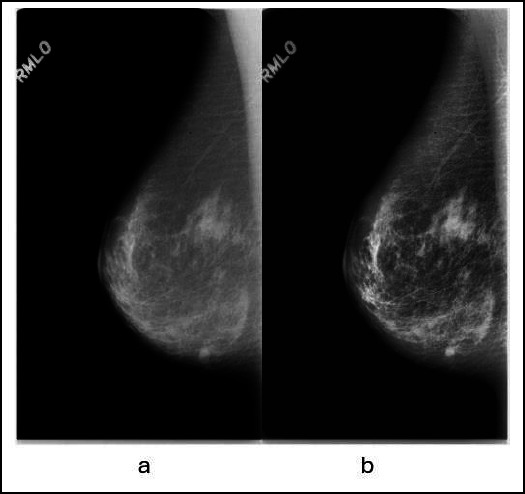
Mammogram images in MLO view, **a**) the image without CLAHE while **b**) the image enhanced by CLAHE.

**Fig. (3):**
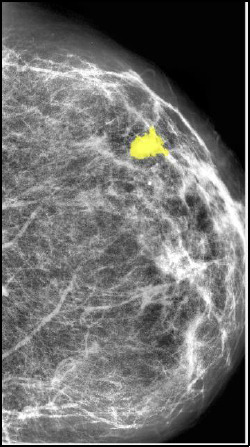
Segmented area in mammography images ACM.

**Fig. (4):**
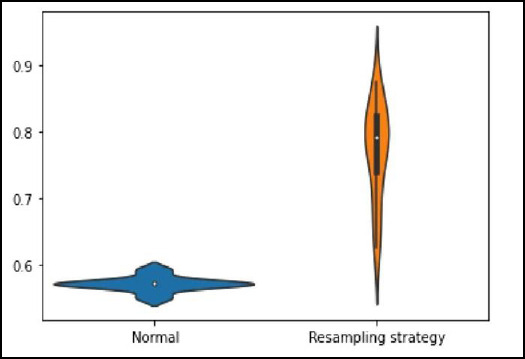
Range of accuracy score for Auto-Sklearn after 15 repetitions.

**Table 1:** Performance matric value between standard and after resampling strategy.

**Evaluation Metric**	**Standard**	**Resampling Strategy**
Accuracy	0.551	0.792
Precision	0.609	0.704
Recall	0.538	0.760
ROC AUC	0.660	0.840

## Data Availability

All data generated or analyzed during this study are included in this published article.

## LIST OF ABBREVIATIONS

ACM: Active Contour Method
AI: Artificial Intelligent
AUC: Area Under Curve
AutoML: Automated Machine Learning
BO: Bayesian Optimization
CBIS: Curated Breast Imaging Subset
CC: Craniocaudal
CNN: Convolutional Neural Networks
CLAHE: Contrast Limited Adaptive Histogram Equalization
GLCM: Gray Level Co-occurance Matrix
DDSM: Digital Database for Screening Mammography
ML: Machine Learning
MLO: Mediolateral Oblique
RF: Random Forest
ROC: Receiver Operating Curve
SVM: Support Vector Machines
XGBoost: Extreme Gradient Boosting
